# Chemical Cleaning of Magnetite Deposits on the Flow Mini-Channels of a Printed Circuit Heat Exchanger in an EDTA-Based Solution

**DOI:** 10.3390/ma15041471

**Published:** 2022-02-16

**Authors:** Jeoh Han, Sang-Ji Kim, Young-Kook Lee, Do-Haeng Hur

**Affiliations:** 1 Materials Safety Technology Development Division, Korea Atomic Energy Research Institute, Daejeon 34057, Korea; jeohhan@kaeri.re.kr; 2 Department of Materials Science and Engineering, Yonsei University, Seoul 03722, Korea; 3 Versatile Reactor Technology Development Division, Korea Atomic Energy Research Institute, Daejeon 34057, Korea; sjkim3@kaeri.re.kr

**Keywords:** printed circuit heat exchanger, fouling, chemical cleaning, magnetite, deposit, corrosion, small modular reactor

## Abstract

The dissolution behavior of magnetite deposited on flow mini-channel surfaces within a printed circuit heat exchanger (PCHE) and the corrosion behavior of a STS 316L PCHE material were investigated in an ethylendiaminetetraacetic acid (EDTA)-based chemical cleaning solution at 93 °C. The fouling in the PCHE was simulated using a water-steam circulation loop system. Most of the magnetite deposits were rapidly dissolved in the early stage of the circulation chemical cleaning. An empirical equation for estimating the dissolution percentage was derived as a function of cleaning time. The PCHE material showed excellent corrosion resistance during the chemical cleaning tests. These results indicate the fouling layers in the PCHEs can be removed efficiently by the chemical cleaning process without concern about base metal corrosion.

## 1. Introduction

A heat exchanger is one of the most important components in the energy production industry, and many studies are underway to increase its heat transfer efficiency. In particular, a shell-and-tube steam generator (SG) consists of thousands of tubes and has the largest volume among the components in a pressurized water reactor (PWR). In addition, SG tubes experience various types of corrosion and mechanical damage due to harsh environments under high-temperature and high-pressure. A rupture of a single tube at operating PWRs may also result in the failure of some neighboring tubes in the tube bundle. As a result, not only heat transfer efficiency, but also safety issues must always be considered together. Recently, there has been a growing interest in printed circuit heat exchangers (PCHEs) of a plate type in an effort to improve the above-mentioned drawbacks [1,2,3,4,5,6,7].

PCHEs are comprised of stacked metal plates in which flow mini-channels are etched photo-chemically. Because of the small hydraulic diameter of the channels, ranging from 300 µm to 2 mm, the heat transfer surface area per unit volume of the heat exchanger drastically increases [8], thereby enabling miniaturization of the heat exchanger itself. In addition, since the alternately stacked cold and hot channel plates are bonded using a diffusion-bonding process, the PCHEs have outstanding mechanical properties under high-temperature and high-pressure conditions. Because of these advantages, there has been a growing interest in the development of PCHEs for application to small modular reactors [9,10]. However, some challenges should also be addressed for the implementation of nuclear PCHEs.

Subcooled water is transformed to steam in the flow channels of the nuclear PCHEs. As a result, non-volatile corrosion products such as magnetite (Fe_3_O_4_) can be deposited on the channel surfaces during the phase transformation, which is referred to as fouling. Severe fouling causes undesirable results in the classic shell-and-tube SGs of PWRs, including thermal performance degradation, thermal hydraulic instability, interference with inspection, and coast-down operation [11,12,13,14,15]. Due to the small flow channel size, PCHEs are expected to be much more vulnerable to fouling compared to conventional SGs. Moreover, some studies have reported that corrosion of the SG tube materials is accelerated by the concentration of aggressive chemical impurities within the pores of the fouling layer [16,17,18,19]. Classic shell-and-tube SG tubing in PWRs can be damaged by a variety of degradation modes, including stress corrosion cracking, intergranular attack, pitting, and fretting-wear [11,12,13,20,21,22,23,24]. To address this, the integrity of the SG tubing has been inspected using eddy current testing (ECT) probes. The thickness of the deposits and clogging of the tube support plate have also been estimated using ECT signals [25,26,27]. In conventional SGs, deposits are removed by mechanical and chemical cleaning processes. The most representative mechanical cleaning method is a sludge lancing, which physically detaches the deposits by inserting high-pressure water jet tools into the tube bundle, regardless of the type of deposit [11,27,28,29]. On the other hand, chemical cleaning is a process to remove the deposits by chemically dissolving using solutions depending on the deposit species, typically iron oxides or copper oxides. At this time, it is important not only to dissolve the deposits efficiently, but also to minimize the corrosion of the base materials [11,27,30,31].

However, these conventional probes and mechanical cleaning tools cannot be applied to PCHEs due to their small channel size and complex channel geometry. Conventional tubes that are damaged due to mechanical or corrosion-related degradation in a PWR SG are repaired by plugging and sleeving, but a PCHE would be replaced with a new one, due to the difficulty of repairing damaged flow channels. Therefore, it is essential to remove corrosion products deposited on the flow channels of nuclear PCHEs using a chemical cleaning process. Nevertheless, no experimental research on the removal of deposits in nuclear PCHEs has been published to date.

The purpose of this work is to investigate the dissolution behavior of magnetite deposited on the flow channel surfaces of a PCHE. A fouling test was first carried out using a water-steam circulation loop system equipped with the PCHE in which water was transformed to steam. The fouled PCHE was then cleaned using a chemical cleaning method. The corrosion of the PCHE material was also evaluated during the chemical cleaning process.

## 2. Experimental Methods

### 2.1. Preparation of the PCHEs for the Fouling Test

**Figure 1:**
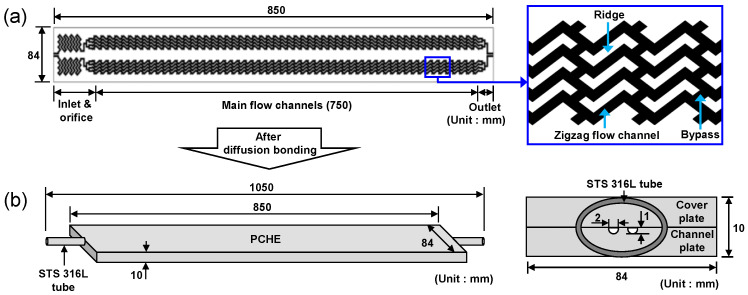
Manufacturing processes and detailed dimensions of the PCHE: (**a**) geometry of the flow channels; (**b**) after the diffusion bonding process.

The PCHEs were manufactured using a photochemical etching and a diffusion bonding process. Flow mini-channels were etched into an STS 316L plate with a size of 84 (W) × 850 (L) × 5 (T) mm. Figure 1a shows the geometry of the flow channels. The flow passages were designed in two symmetrical shapes in the longitudinal direction, and one unit flow passage consisted of a feedwater inlet, an orifice, main flow channels with a zigzag-shape, and a steam outlet. All cross-sections of the etched flow channels had a semicircular shape with a diameter and height of approximately 2 mm and 1 mm, respectively. The channel plate was then diffusion-bonded without inserting an interlayer with an STS 316L cover plate with the same dimensions as the channel plate at a temperature of 900 °C and a pressure of 10 MPa for 60 min, which corresponds well with similar diffusion bonding parameters for this material [32]. Subsequently, STS 316L tubes, which will serve as a header, were welded to the inlet and outlet of the PCHE. A schematic diagram of the finally manufactured PCHE is shown in Figure 1b.

### 2.2. Fouling Test

**Figure 2:**
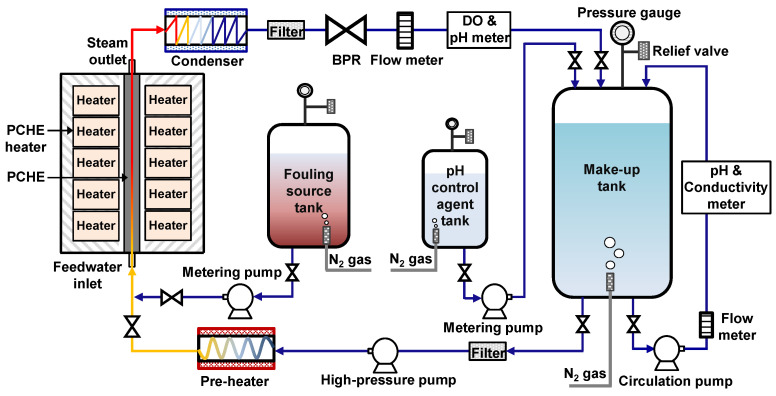
Schematic diagram of the circulation loop system for the PCHE fouling test.

Figure 2 is a schematic diagram of a water-steam circulation loop system used for the PCHE fouling tests. The fouling tests were performed for 500 h each using two fresh PCHEs under the thermal hydraulic parameters and water chemistry conditions given in Table 1. These thermal hydraulic parameters correspond with the operating conditions of a small modular reactor, named SMART (System-integrated Modular Advanced ReacTor) [33,34,35].

Deionized water with a pH of 9.8 ± 0.2 at 25 °C was used as feedwater, and the pH was maintained using ammonia. The feedwater was deaerated by blowing high-purity nitrogen gas in a make-up tank, and thus dissolved oxygen (DO) was controlled at less than 5 µg/L during the test. This is a typical secondary water chemistry for PWRs [30]. The feedwater stored in the make-up tank was heated to about 230 °C via pre-heaters and fed into the PCHE at a flow rate of 340 mL/min. Commercial PCHEs which are currently used in various fields are composed of alternately stacked multi-hot and cold plates in which flow channels are etched. Therefore, heat is exchanged through each flow channel plate without phase transformation. Unlike commercial PCHEs, in nuclear PCHEs the feedwater flowing through cold channel plates undergoes a phase transformation. However, the PCHE fabricated in this work had only a single cold channel plate for the water-to-steam change, as shown in Figure 1. Therefore, heat fluxes were supplied to both sides of the PCHE main flow channel surfaces using ten electric heaters to generate superheated steam of 303.5 °C. The superheated steam was transformed to the single-phase water state through a condenser and returned into the make-up tank. Iron acetate was artificially injected as a fouling source into the water-steam circulation environment, so that the iron concentration of the feedwater flowing into the PCHE inlet was a constant 100 µg/L until the test was completed.

### 2.3. Chemical Cleaning of Deposits Accumulated in the PCHEs

**Figure 3:**
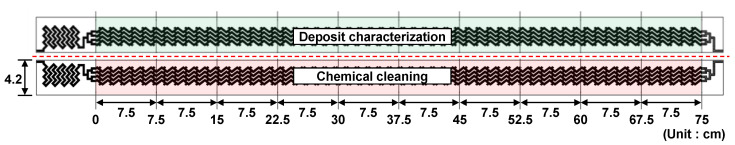
Drawing for sectioning the PCHE.

After the first fouling test, the PCHE was bisected along the centerline between the symmetric flow channels, and then each main flow channel region was cut widthwise into ten equal zones as shown in Figure 3. One flow channel was used to identify the morphology, chemical composition, and crystal structure of the fouling deposits to determine appropriate the chemical cleaning conditions. The other channel was used to quantify the amount of deposits removed from each zone by chemical cleaning.

Deposits are accumulated on the narrow flow channel surfaces in the PCHE. Therefore, the cover plate part was first carefully eliminated by machining to expose the fouled surfaces of the flow channels. This enabled us to analyze deposits accumulated to the etched channel surfaces microscopically and spectroscopically. An optical microscope was used to visually observe the deposits on the channel surfaces. The detailed surface appearance and size of the deposits were also investigated using a high-resolution scanning electron microscope (SEM) with an acceleration voltage of 10 kV. Specimens for scanning transmission electron microscope (STEM) observation were fabricated using a focused ion beam (FIB) technique and then the chemical compositions of the deposits were analyzed using an energy dispersive spectroscope (EDS) in the TEM. Deposit particles were scraped from the exposed channel surfaces using a plastic knife and collected to determine their crystal structure using a high-resolution X-ray diffractometer (XRD) with a Cu-Kα radiation (λ = 1.54056 Å) at a scan rate of 0.01 °/s.

**Figure 4:**
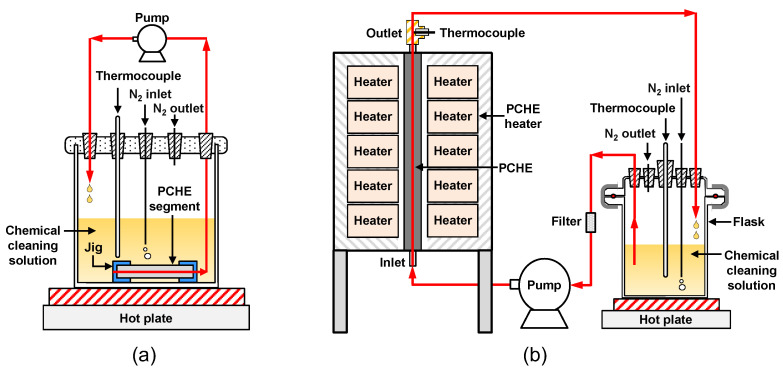
Schematic diagrams of the chemical cleaning apparatus: (**a**) circulation cleaning for a PCHE segment; (**b**) circulation cleaning for a full-length PCHE.

The chemical cleaning tests were carried out using the following solution according to the Electric Power Research Institute/Steam Generator Owners Group (EPRI/SGOG, Palo Alto, CA, USA) cleaning process: 20 wt.% diammonium ethylendiaminetetraacetic acid ((NH_4_)_2_EDTA), 1 wt.% hydrazine (N_2_H_4_), 1 wt.% corrosion inhibitor (CCI-801). The pH of the solution was adjusted to 7.0 at 25 °C using ammonia solution (NH_4_OH). This EDTA-based solution was developed for iron oxide dissolution by the EPRI and has been implemented in many PWR SGs [11,27,36,37,38] around the world. The CCI-801 is manufactured by Petrolite Corporation (Saint Louis, MO, USA), and the exact ingredients of the product cannot be known due to confidentiality. However, commonly used corrosion inhibitors form a corrosion-resistant film on the metal surfaces, resulting in a decrease of the corrosion rate [39,40,41,42,43]. The chemical cleaning was performed in two steps: first, each PCHE segment was immersed in a static reaction cell containing the chemical cleaning solution at 93 °C for 12 h; in the second step, specially made jigs were mounted to both ends of the PCHE segment, so that the cleaning solution could be circulated through the flow channel holes via a circulation pump at a flow rate of about 30 mL/min, as depicted in Figure 4a. Under this condition, chemical cleaning further proceeded at 93 °C for 12 h. The solution was refreshed before the second step cleaning. After the cleaning in each step, the concentrations of iron ions dissolved in the chemical cleaning solutions were analyzed using inductively coupled plasma–atomic emission spectroscopy (ICP-AES).

On the other hand, after the second fouling test, the full-length PCHE was used for chemical cleaning without sectioning. As shown in Figure 4b, the chemical cleaning solution was fed into the PCHE inlet at a flow rate of about 30 mL/min by a circulation pump and the temperature of the solution was maintained at 93 °C by the heaters and a hot plate. The chemical cleaning was performed for a total of 42 h, and sampling was periodically conducted to measure the iron concentrations dissolved in the cleaning solutions. After sampling, the cleaning solution was always replaced with a new one.

### 2.4. Corrosion Test

**Figure 5:**
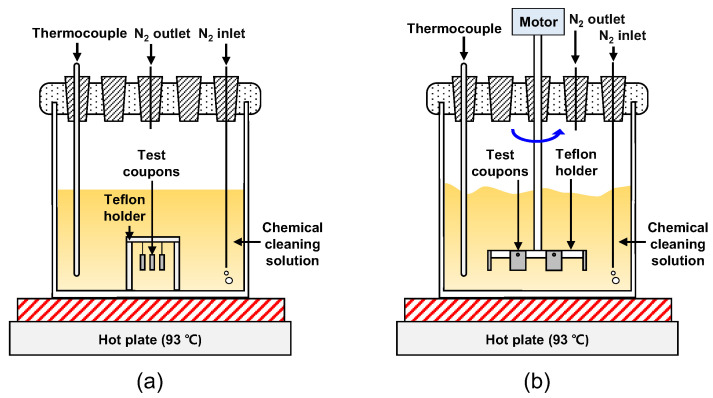
Schematic diagrams of the corrosion test apparatus under (**a**) the stagnant condition; (**b**) the flowing condition.

The corrosion of the PCHE material, which may occur during chemical cleaning, was evaluated from the weight change of corrosion coupons in the EDTA-based solution at 93 °C. All of the coupons were polished using 100 grit silicon carbide paper to simulate the actual surface roughness of the PCHE flow channels (average roughness (R_a_) = 1.38 µm). As shown in Figure 5, the tests were conducted under two different conditions. For the stagnant immersion test, corrosion coupons were placed on a Teflon holder. The test under the flowing condition was designed to simulate corrosion during the solution circulating cleaning. Coupons were mounted on a Teflon holder with a regular dodecagonal shape and the holder was rotated using a motor. From the angular velocity, the flow rate of the solution at the surface of the specimens was controlled to be 6 m/min, analogous to that of the circulating cleaning for the full-length PCHE. The weight change was periodically measured using an electrical balance with a readability of 10 µg. The surfaces of the coupons were also examined using SEM to compare the corrosion behavior before and after the chemical cleaning process.

## 3. Results and Discussion

### 3.1. Characterization of Deposits

**Figure 6:**
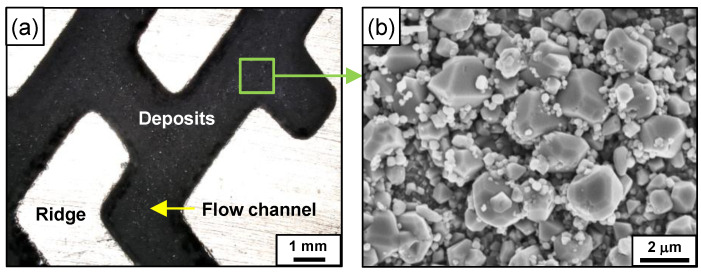
Images of the PCHE flow channels after the fouling test: (**a**) optical and (**b**) SEM.

Figure 6 shows optical and SEM images of the flow channel surfaces after the fouling test. The surfaces of the flow channels were entirely covered with black-colored deposits. The deposits consisted of polyhedral particles with various sizes from tens of nanometers to about 2 μm and were accumulated so densely that the original surface of the flow channels could not be observed.

**Figure 7:**
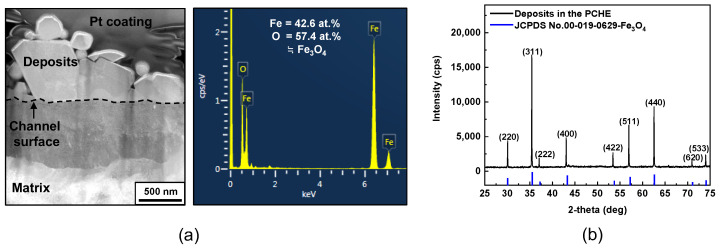
Chemical composition and crystal structure of the deposits within the PCHE: (**a**) STEM image and EDS spectra, and (**b**) XRD patterns.

Particles deposited on the channel surface were milled to make STEM specimens using FIB. However, it was very challenging to extract the TEM foils from the grooved and narrow channel surface. Figure 7a shows the STEM images and EDS analysis results of the deposits. It can be seen that some particles were deposited on the matrix. The result of the EDS point analysis revealed that the chemical composition of the deposit particles had a stoichiometry corresponding to that of magnetite.

Figure 7b shows the X-ray diffraction patterns of the deposits collected from the flow channel surfaces. The Miller indices for each peak indicated that the particles had a cubic spinel structure in the Fd-3m space group. Furthermore, all the Bragg planes coincided with the reference XRD patterns of crystalline magnetite with a cubic spinel structure, and no other distinct diffractions were observed. The chemical composition, morphology and crystal structure of the deposits accumulated within the PCHE were similar to the actual SG tube deposits in PWRs [44,45,46,47]. Therefore, the above results provide a solid basis for the selection of the EDTA-based chemical cleaning solution.

### 3.2. Dissolution Behavior of Magnetite Deposits within the PCHEs

**Figure 8:**
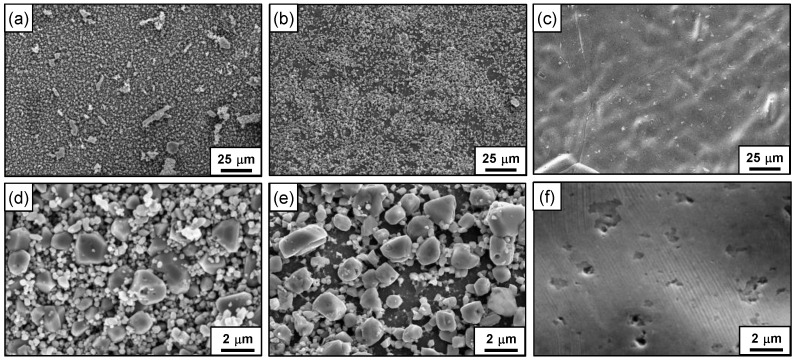
SEM micrographs of the PCHE flow channel surfaces: (**a**,**d**) before chemical cleaning; (**b**,**e**) after the first step cleaning for 12 h; (**c**,**f**) after the second step cleaning for 12 h.

Figure 8 shows the SEM micrographs of the flow channel surfaces of the PCHE segments before and after the two-step chemical cleaning process. After the first step cleaning, some small particles dissolved and the sharp facets of the particles became relatively roundish, but significant amounts of deposits still remained undissolved on the channel surfaces as shown in Figure 8b,e. After the second step cleaning, the remaining deposits had almost disappeared, so that the original channel surfaces were clearly exposed, as shown in Figure 8c,f. It is assumed that the magnetite dissolution and removal proceeded through reactions (1) and (2) [48]. One molecule of magnetite contains one ferrous ion and two ferric ions. Therefore, magnetite is reductively dissolved by hydrazine according to reaction (1), and then the dissolved ferrous ions are chelated by EDTA according to reaction (2).

2Fe_3_O_4_ + N_2_H_4_ + 4H_2_O → 6Fe^2+^ + 12OH^−^ + N_2_ (1)

Fe^2+^ + (NH_4_)_2_EDTA → Fe–EDTA + 2NH_4_^+^ (2)

**Figure 9:**
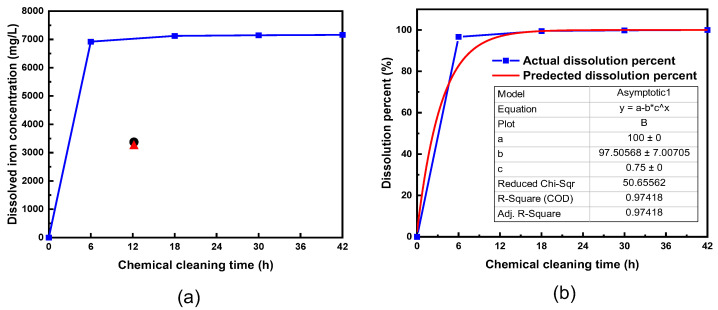
(**a**) Change in the iron concentrations dissolved from the full-length PCHE with cleaning time under the circulation condition. The solid black circle and red triangle denote the iron concentration dissolved in the first and second step, respectively. (**b**) Derivation of the empirical equation of dissolution percent using the dissolved iron concentration change curve with time.

Figure 9a shows the magnetite dissolution behavior of the full-length PCHE under the circulation cleaning condition. The magnetite dissolution reaction proceeded quickly in the early stage of chemical cleaning, and approximately 96% of the total deposits were dissolved in 6 h. The dissolved iron concentration was no longer increased after a cumulative cleaning time of 30 h, indicating the magnetite deposits were completely dissolved within 30 h.

The magnetite dissolution at each time during the PCHE full-length cleaning can be expressed as an asymptotic equation. An empirical equation for the magnetite dissolution was derived from the data in Figure 9a, and is shown in Figure 9b. The blue solid squares represent the dissolution percent at each cleaning time that is calculated in Figure 9a, while the red line is the asymptotic fitting line of the blue squares. The fitting yielded the following formula.

(3) $$\mathrm{Predicted}\;\mathrm{dissolution}\;\mathrm{percent}\;\left(\%\right)=100-97.5\left(0.75^{t}\right)\;(R^{2}=0.974)$$

From this formula, the percent of magnetite deposit dissolution can be estimated as a function of cleaning time (*t*). However, magnetite dissolution can be relative depending on the following factors: the inventory of magnetite deposits, EDTA concentration and the temperature of the cleaning solution, and the circulation rate of the cleaning solution. Therefore, it is important to identify the total mass and distribution of deposits accumulated in a PCHE using non-destructive methods prior to chemical cleaning. In future studies, it will be meaningful to derive optimal cleaning conditions using factors related to various magnetite dissolution behavior. Meanwhile, chemical cleaning should be applied efficiently and safely to PCHEs. That is the reason the traditional SG cleaning process is monitored by analyzing solution samples and corrosion coupons periodically. As a result, endpoint determination of cleaning is important. From the dissolution behavior described above, the best time for cleaning would be between 6 h and 12 h.

**Figure 10:**
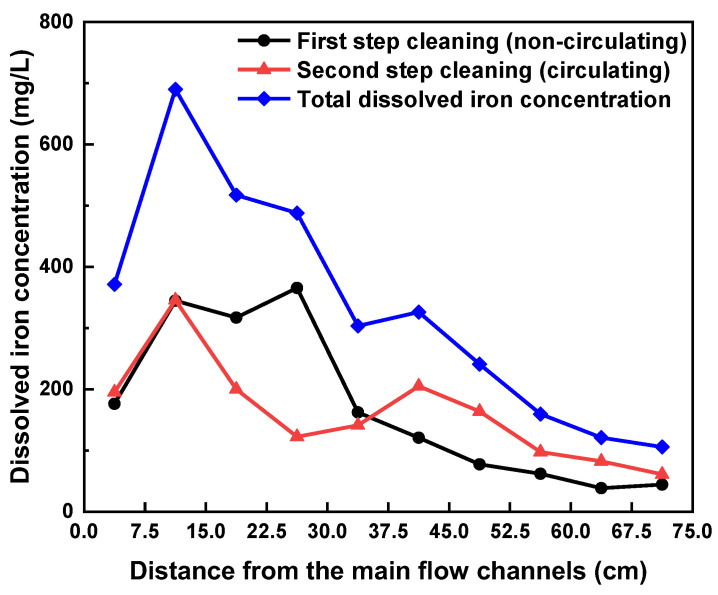
Iron concentrations dissolved from the main flow channels during the two-step cleaning process.

Figure 10 shows the dissolution behavior of the magnetite deposits depending on each zone of the main flow channels during the two-step cleaning process. To compare the cleaning efficiency of each cleaning step, the iron concentration dissolved in each step was obtained by summing up the iron concentration of each zone, which yielded 3419 mg/L in the first step for 12 h and 3230 mg/L in the second step for 12 h. Please note that the iron concentration in Figure 10 resulted from only one of the two symmetrical main flow channels. As a result, the above two values were obtained by multiplying the sum of the iron concentration of each zone by two. At first glance, these values may lead to misjudgment that the dissolution kinetics of deposits are similar to each other in each step. For comparison, the iron concentrations dissolved in the first and second steps were superimposed in Figure 9. This clearly indicates that the iron concentration dissolved under the static immersion cleaning condition (i.e., the first step) is not even half of what it is expected from the full-length circulation cleaning. Dissolution is a process by which a solute in a solid phase dissolves in a solvent to form a solution. Thus, the rate at which a solid substance dissolves is a function of the collision frequency between the substance and the solvent and can be increased by the following methods: reducing the particle size, increasing the temperature, and increasing the mixing or stirring rate [49,50,51,52]. Accordingly, when a PCHE segment was statically immersed in the chemical cleaning solution, collision between deposits and solvent may not actively occur, resulting in a significant decrease in the dissolution and chelation rate. However, the circulation of the cleaning solution facilitates not only the supply of the fresh solution inside the small and complex channels but also the removal of the iron-EDTA chelates from the channels, thereby resulting in a faster dissolution of deposits than under the static immersion cleaning condition. As a result, it is expected that the deposits remained after the first step had already been dissolved before the second step cleaning was terminated. This inference can also be supported by the result that 96% of the total deposits were dissolved in 6 h during the full-length circulation cleaning, as shown in Figure 9a.

**Figure 11:**
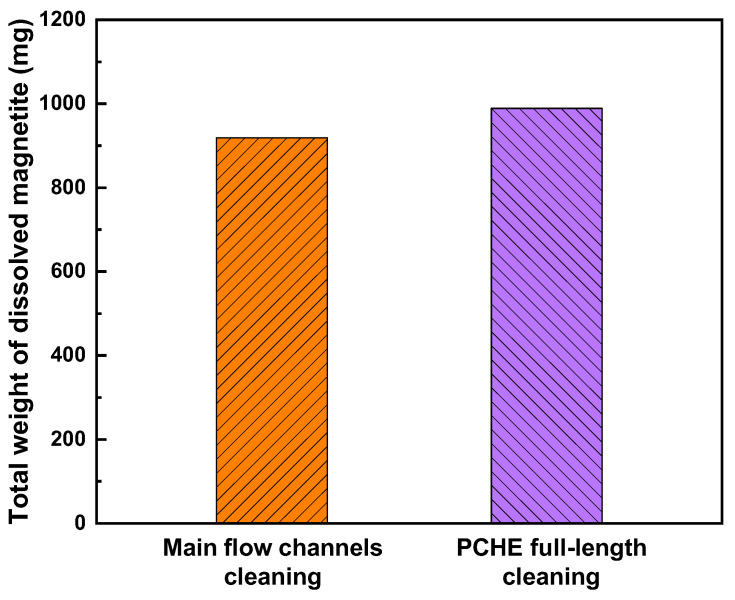
Total weight of magnetite dissolved from each cleaned PCHE.

The iron concentration in the cleaning solution results from the dissolution of magnetite deposits. Therefore, the amount of magnetite deposits can be calculated using the dissolved iron concentration and solution volume. Figure 11 shows the total weight of the magnetite dissolved during the full-length cleaning (Figure 9a) and main flow channel cleaning (Figure 10), respectively. The deposit amount removed from the full-length PCHE was approximately 7% greater than that from the sectioned channels, and the reason for that can be discussed as follows: despite all of the precautions, a proportion of the deposited particles were inevitably detached from the channel surfaces during the PCHE sectioning process. In addition, the value of the sectioned channels was obtained only from the segments of the main flow channels, except for the PCHE inlet and outlet sections (Figure 3).

### 3.3. Corrosion Behavior of the PCHE Base Material

**Figure 12:**
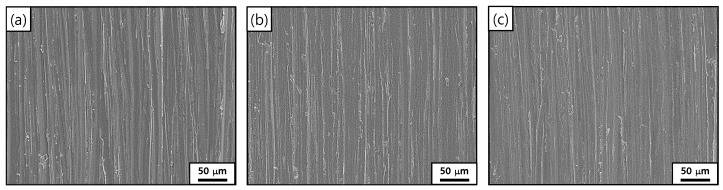
SEM micrographs of the STS 316L corrosion coupon surfaces: (**a**) before the corrosion test; (**b**) after the corrosion test under the stagnant condition for 42 h; (**c**) after the corrosion test under the flowing condition for 42 h.

Figure 12 shows SEM micrographs of the STS 316L coupons before and after the corrosion tests in the EDTA-based solutions for 42 h. Abrasive marks produced by silicon carbide paper during the specimen preparation were intact after the exposure to the solutions, regardless of the solution flow. This indicates that the general corrosion rate of STS 316L is significantly slow during the chemical cleaning process. In addition, localized corrosion such as pitting was not observed. This means that STS 316L had a significantly low corrosion rate in the EDTA-based cleaning solution, and the circulation of the cleaning solution did not affect corrosion.

**Figure 13:**
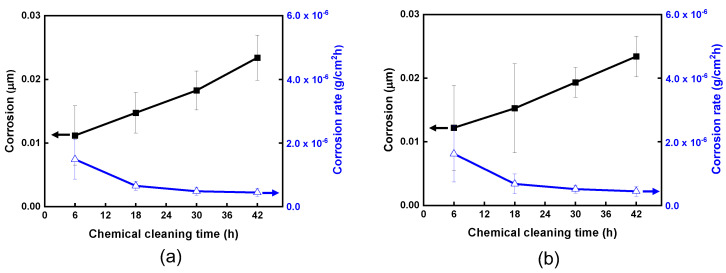
Corrosion of the STS 316L coupons under (**a**) the stagnant condition and (**b**) the flowing condition.

Figure 13 shows uniform corrosion penetration of the STS 316L coupons in the EDTA-based solutions. Corrosion coupons were weighed before and after the corrosion test. The corrosion of the coupons was calculated using the following equation.

(4) $$\mathrm{Corrosion}\;(\mathrm{cm})=\frac{\mathrm{Weight}\;\mathrm{loss}\;\left(g\right)}{\mathrm{Density}\;\mathrm{of}\;\mathrm{the}\;\mathrm{coupon}\;\left(g/{\mathrm{cm}}^{3}\right)\times \;\mathrm{Surface}\;\mathrm{area}\;\left({\mathrm{cm}}^{2}\right)}$$

The corrosion increased with cleaning time, but the values were extremely low, at about 0.02 μm, even after 42 h. It was also confirmed that the corrosion was not affected by the solution flow. According to the EPRI guidelines [36], the allowable corrosion limits are 254 μm for low alloy steels and 6 μm for Alloy 600 tubing in the classic PWR SGs during a chemical cleaning period. This indicates that the chemical cleaning method using an EDTA-based solution can be implemented for fouled PCHEs within well-controlled base metal corrosion. Furthermore, it is confirmed that all of the iron ions dissolved in the cleaning solution resulted from the dissolution of the deposits formed on the PCHE channel surfaces during the cleaning process.

## 4. Conclusions

The dissolution behavior of magnetite deposits within PCHEs and the corrosion of the PCHE material were investigated when chemical cleaning was performed with an EDTA-based solution, and the following conclusions were obtained.

(1) The deposits on the flow channels of the PCHE were black-colored polyhedral-shaped particles, which had a stoichiometry and a crystalline structure corresponding to that of magnetite. The characteristics of the PCHE deposits were analogous to the deposits which accumulate in the shell-and-tube SGs of PWRs. These results provided a solid basis for selecting the EDTA-based chemical cleaning process to remove the magnetite deposits within the PCHEs.
(2) The magnetite deposits attached on the channel surfaces of the PCHEs were easily removed by the 20% EDTA-based chemical cleaning solution. In particular, most of the magnetite deposits were dissolved in the early stage of the full-length circulation cleaning, and all of the deposits were completely removed within 30 h. From the full-length cleaning process, an empirical equation of magnetite dissolution percent with cleaning time was derived.
(3) The surfaces of the STS 316L coupons were uniform even after the 42-h corrosion tests in the EDTA-based solution, and localized corrosion such as pitting was not observed. In addition, the corrosion values were significantly lower than the allowable corrosion limit of various SG materials specified in the guideline, regardless of the circulation of the cleaning solution. These results indicate that the fouling layer within PCHEs can be effectively removed by the EDTA-based chemical cleaning process without concern about base material corrosion.
(4) For future research, it is necessary to quantify various factors affecting the dissolution behavior of the deposits. Chemical cleaning is a follow-up measure that causes economic and operational burden for fouled PCHEs. Therefore, proactive methodologies for mitigating the generation and transport of corrosion products are essential for future works.

## Figures and Tables

**Table 1:** Thermal hydraulic parameters and water chemistry conditions for the fouling test.

Thermal Hydraulic Parameter				Water Chemistry Condition		
Feedwater inlet temperature	Steam outlet temperature	Steam outlet pressure	Flow rate	[Fe] concentration in feedwater	Dissolved oxygen	pH_25 °C_
230 °C	303.5 °C	57.6 bar	340 mL/min	100 µg/L	<5 µg/L	9.8

## Data Availability

Not applicable.
